# HAGP: A Heuristic Algorithm Based on Greedy Policy for Task Offloading with Reliability of MDs in MEC of the Industrial Internet

**DOI:** 10.3390/s21103513

**Published:** 2021-05-18

**Authors:** Min Guo, Xing Huang, Wei Wang, Bing Liang, Yanbing Yang, Lei Zhang, Liangyin Chen

**Affiliations:** 1 School of Computer Science & School of Software Engineering, Sichuan University, Chengdu 610065, China; guomin@stu.scu.edu.cn (M.G.); 2017223045118@stu.scu.edu.cn (X.H.); wang.david.wei@stu.scu.edu.cn (W.W.); 2019223049277@stu.scu.edu.cn (B.L.); yangyanbing@scu.edu.cn (Y.Y.); zhanglei@scu.edu.cn (L.Z.); 2 School of Mathematics and Computer Science, Northwest Minzu University, Lanzhou 730050, China; 3 Institude for Industrial Internet Research, Sichuan University, Chengdu 610065, China

**Keywords:** mobile edge computing (MEC), task offloading, reliability, optimization, Industrial Internet

## Abstract

In the Industrial Internet, computing- and power-limited mobile devices (MDs) in the production process can hardly support the computation-intensive or time-sensitive applications. As a new computing paradigm, mobile edge computing (MEC) can almost meet the requirements of latency and calculation by handling tasks approximately close to MDs. However, the limited battery capacity of MDs causes unreliable task offloading in MEC, which will increase the system overhead and reduce the economic efficiency of manufacturing in actual production. To make the offloading scheme adaptive to that uncertain mobile environment, this paper considers the reliability of MDs, which is defined as residual energy after completing a computation task. In more detail, we first investigate the task offloading in MEC and also consider reliability as an important criterion. To optimize the system overhead caused by task offloading, we then construct the mathematical models for two different computing modes, namely, local computing and remote computing, and formulate task offloading as a mixed integer non-linear programming (MINLP) problem. To effectively solve the optimization problem, we further propose a heuristic algorithm based on greedy policy (HAGP). The algorithm achieves the optimal CPU cycle frequency for local computing and the optimal transmission power for remote computing by alternating optimization (AP) methods. It then makes the optimal offloading decision for each MD with a minimal system overhead in both of these two modes by the greedy policy under the limited wireless channels constraint. Finally, multiple experiments are simulated to verify the advantages of HAGP, and the results strongly confirm that the considered task offloading reliability of MDs can reduce the system overhead and further save energy consumption to prolong the life of the battery and support more computation tasks.

## 1. Introduction

In the Industrial Internet, computing- and power-limited mobile devices (MDs) related to the production process can hardly support computation-intensive and time-sensitive applications, such as smart sensing for production environments, healthcare monitoring of production machines, and smart transportation of production materials [1,2,3,4]. At the same time, with the massive amount of MDs connected to the Industrial Internet, security is also an urgent problem that needs to be solved [5]. Mobile edge computing (MEC) is hence considered as a promising solution for those issues through processing application requests approximately close to the MDs [6,7,8]. When computation tasks are offloaded to the edge server, extra transmission delay will also be generated, except for inherent processing latency and energy consumption. Therefore, the trade-off between latency and energy consumption is not only one of the main goals for task offloading but also an important metric for evaluating the performance of an MEC system [9,10,11]. However, task offloading decisions in MEC are easily affected by many uncertain factors, such as unstable mobile wireless channels, resulting in unpredictable latency and more energy consumption caused by unnecessary task re-transmission, which may seriously degrade the system performance [12,13]. Moreover, complex task offloading schemes or mechanisms will also consume precious resources, e.g., battery power in MDs, which will further influence the system performance [14,15,16]. Therefore, ensuring reliable task offloading in MEC is a must and necessary requirement in realistic Industrial Internet application scenarios.

With the widespread popularity of MEC, there are some seminal works considering the reliability for task offloading in MEC systems [17,18,19,20,21]. In particular, to handle the uncertain communication condition for transmitting the data and instructions required by computation tasks, a joint optimization scheme was proposed in [19] to achieve the trade-off between latency and reliability in task offloading, but it ignores finite computing power on both MDs and edge servers in real applications. To guarantee the reliability of both computing modes, a novel optimization problem of computation and transmission power in task offloading was presented in [20], which is subjected to the latency and reliability constrained by task queue length violations on the MD and server side. However, its experiment results show that the reliability is closely related to the task arrival rates, rather than the computing capability or battery power of MDs. Considering the importance of MDs for task offloading in MEC, an energy-efficient task offloading scheme was studied in [21], which satisfies the reliability existing in local and offloading schemes because of the uncertain computing power and transmission rate, respectively. However, it is still questionable for the reliability of MDs measured by the battery level. In fact, when the MD is reliable, the task would be processed by the computing mode with the optimal objective or dropped actively by the MD with a penalty. Otherwise, the task will be disrupted and discarded by the exhausted battery power, which will consume more execution overhead compared with the case of a reliable MD [22,23,24]. Therefore, to make the task offloading scheme more suitable for the actual production environment, the limited energy power is an important constraint that needs to be met.

To address this issue, this paper focuses on the reliability of MDs when making the offloading decision in an MEC system. In more detail, we define the reliability of MDs with the residual battery power after the completion of computation tasks according to [25,26]. Subsequently, we formulate an optimization problem by minimizing the weighted sum of the process latency and energy consumption and then propose a heuristic algorithm based on greedy policy, namely, HAGP. Finally, the results obtained through extensive simulation experiments show that the task offloading scheme with reliability of MDs in MEC will consume a lower system overhead and further prolong the battery lives of MDs, and vice versa, which is consistent with the actual situations.

In a nutshell, the main contributions of this paper are summarized as follows:

1. We consider a computation task offloading scenario with an edge server and multiple heterogeneous MDs, where a different type of computation task is randomly requested by each MD, and the computing power of the edge server is constrained by the number of channels existing between MDs and the edge server, by which they can exchange data and information.
2. We define the reliability of MDs as the residual energy of MDs after completing a computation task and formulate the problem of computation task offloading in this scenario as a mixed integer non-linear programming (MINLP) problem.
3. We solve the problem with alternating optimization (AP) methods and, based on these, propose and design a heuristic algorithm, HAGP, to make decisions for processing computation tasks on MDs, which would minimize the system overhead consisting of the weighted sum of the process time delay and energy consumption.
4. We conduct extensive simulation experiments and theoretically analyze the results to verify the performance and confirm the advantages of HAGP by comparing with several baseline algorithms.

The structure of this paper is organized as follows. Firstly, the system models, including the networking model, computation model, communication model, and reliability model, are built in Section 2. Then, the definition of the system overhead and optimization problem is formulated in Section 3. Section 4 provides the solving process for the optimization problem and presents the algorithm designed to obtain the offloading scheme. Subsequently, Section 5 shows the simulation results and verifies the advantages of the proposed algorithm by comparing with several classical baseline algorithms. Finally, the conclusion is in Section 6.

## 2. System Models

This section mainly describes the formulation of different models and builds the optimization problem that will be solved in the subsequent part of the article. Firstly, we define the reliability of MDs with residual energy after the execution of a computation task. Subsequently, we describe the MEC system model used in this paper, i.e., task offloading with reliability of MDs in an MEC system of the Industrial Internet. Then, both the local computing model and remote computing model are represented. After that, the overhead of the system is defined to evaluate the offloading decision. Finally, the optimization problem is formulated and solved.

### 2.1. Overall System Model

**Figure 1:**
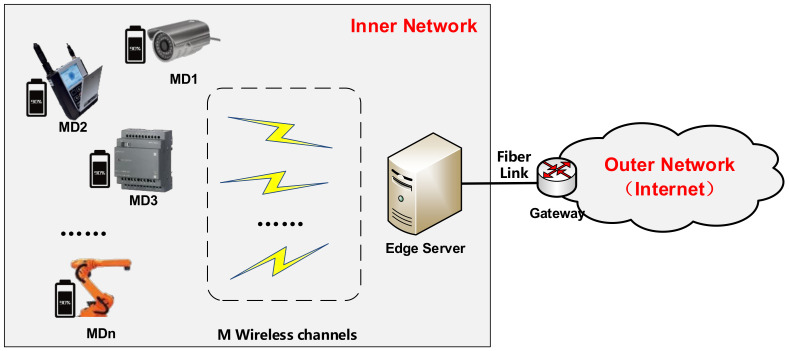
The scenario of task offloading with reliability of MDs in MEC of the Industrial Internet.

As shown in Figure 1, the overall system model consists of N heterogeneous MDs with different computing powers and battery capacities and an edge server which could be a micro-cell or small-cell base station. For manufacturers in the Industrial Internet, the more MDs that an edge server can serve with limited computing resources, the more economic benefits they will obtain [27,28]. Moreover, the distance between each MD and the edge server can be represented by $d_{i}$, which will cause the differences in channel gains existing during the data transmission. Additionally, all the MDs can exchange data and information with the edge server through one of the M wireless channels. Assume the channels have an individual identity distribution (i.i.d), i.e., the status of channels does not change during one offloading. For convenience, some important symbols adopted in this paper and their description are listed in Table 1.

Consider the computation-intensive application tasks requested by $MD_{i},i\in N$ are represented by $T_{i}=\left(S_{i},D_{i}\right)$, where $S_{i}$ is the size of the computation task with the maximum value of $S_{max}$, including the instructions and dataset requested for task processing (in bit), and $D_{i}$ is the deadline of the computation task (in ms), which means that the computation task must be completed within the specified time. Here, we assume that there is no buffer to queue the computation tasks, which means that computation tasks must be processed in time. The computation tasks are atomic, meaning that they can be either processed locally or offloaded to the edge server for processing, which can be denoted as $I_{i}^{l}=1,I_{i}^{r}=1$. Additionally, if the battery power of the MD is too low to support the execution, or the process latency exceeds the deadline of a computation task, it can be viewed as a fail, namely, $I_{i}^{f}=1$. In this case, the penalty will be added. Thus, the indicator $I=(I_{i}^{l},I_{i}^{r},I_{i}^{f})$ is denoted, which represents the offloading decision for the computation task requested by $MD_{i}$. According to the definition, the offloading decision should be satisfied by

(1) $$\left|I\right|=\sum_{s}\left|I_{i}^{m}\right|\;m\in \left\{l,r,f\right\},i\in N$$

### 2.2. Local Computing

Assume that the number of the CPU cycle frequency required for $MD_{i}$ to process one bit of data is *Q*, which would vary with different applications [29]. In consequence, the number of the CPU cycle frequency required to complete the computation task $T_{i}$ is $S_{i}Q$, and the latency $L_{i}^{l}$ during the computation task processed at $MD_{i}$ can be obtained by

(2) $$L_{i}^{l}=\frac{S_{i}\cdot \;Q}{f_{i}}\;i\in N\;$$

where $f_{i}$ represents the computing frequency of $MD_{i}$ to process the computation task $T_{i}$ locally. Moreover, according to dynamic voltage and frequency scaling (DVFS), the MDs can work with different CPU frequencies ranging from 0 to $f_{i}^{max}$, that is, $f_{i}\in \left[0,f_{i}^{max}\right]$.

Correspondingly, the energy consumed for local computing is

(3) $$E_{i}^{l}=\kappa \;S_{i}Qf_{i}^{2}\;i\in N\;$$

where κ is the coefficient of switching capacitance, decided by the chip manufacturer [30], and the value is usually $10^{-28}$ [31].

### 2.3. Remote Computing

Remote computing in this paper refers to the computation task processed by the edge server near MDs, which needs to transmit data and instructions through wireless channels between them. Therefore, in this computing model, we firstly introduce the communication model [32].

#### 2.3.1. Communication Model

In this paper, there are *M* orthogonal channels between MDs and the edge server, which means the edge server can serve *M* MDs simultaneously at any time. Moreover, the interference among the occupied channels is ignored. Therefore, from Shannon’s theorems [33], the uplink rate for transmitting data and instructions of the computation tasks is

(4) $$v_{i}=\omega \log_{2}\left(1+\frac{h_{i}\cdot \;p_{i}}{\sigma }\right)\;i\in N$$

where ω represents the bandwidth for transmitting, and σ refers to the background noise whose value is $10^{-13}$ in this paper. Furthermore, $p_{i}$ is the power efficiency of $MD_{i}$ to transmit the computation task, and $h_{i}$ represents the channel gain of $MD_{i}$ and obeys an exponential distribution whose unity mean is $g_{0}d_{i}^{-4}$, in which $g_{0}$ is the path loss constant with a value of $10^{-4}$, and $d_{i}$ is the distance between $MD_{i}$ and the edge server, following a uniform distribution with (0,50).

#### 2.3.2. Remote Computing

There are three phrases that should be experienced by a computation task when the MD chooses remote computing. These contain the uplink transmission of the primal computation task, processed by the edge server, and the return of the output results. However, in this paper, the computing capacity of the edge server is limited by the number of wireless channels between MDs and the edge server. In addition, since the output size of the computation task is much smaller than the size of input data, the latency for remote computing is mainly considered as the uplink transmission latency, ignoring the executing latency and downlink transmission latency, i.e., offloading decisions of MDs should be satisfied by

(5) $$\sum_{i=1}^{N}1_{\left\{I_{i}^{r}=1\right\}}\leq M\;i\in N$$

Here, $1_{\left\{A\right\}}$ is a binary function with $1_{\left\{A\right\}}=1$ if A is true and $1_{\left\{A\right\}}=0$ otherwise. Additionally, based on the communication model described in (4), we can obtain the latency of remote computing by

(6) $$L_{i}^{r}=\frac{S_{i}}{v_{i}}\;i\in N$$

In this case, this work focuses on MDs and the edge server providing a service to computation tasks without consuming the energy of MDs; hence, the energy consumption of remote computing is mainly caused by the transmission process. Since the transmission power $p_{i}$ (in w) is given, the energy consumption of remote computing can be formally expressed as

(7) $$E_{i}^{r}=p_{i}\cdot \;L_{i}^{r}\;i\in N$$

where $p_{i}$ represents the energy consumption per unit of time.

### 2.4. Process Latency Model

As a performance metric of processing computation tasks, process latency can be summarized as follows according to different offloading decision and computation models.

(8) $$L_{i}\left(I_{i},f_{i},p_{i}\right)=I_{i}\cdot \left(L_{i}^{l},L_{i}^{r},L_{i}^{f}\right)=I_{i}^{l}L_{i}^{l}+I_{i}^{r}L_{i}^{r}+I_{i}^{f}L_{i}^{f}$$

where $L_{i}^{f}$ is the latency penalty when the computation task is failed, caused by the unreliability of $MD_{i}$, which is a constant equal to the maximum deadline of computation tasks.

### 2.5. Energy Consumption Model

Assume $B_{i}$ is the initial energy of $MD_{i},i\in N$, which are different values due to the heterogeneity of MDs. According to both of the models above, the energy consumption required to complete a computation task can be represented by

(9) $$E_{i}\left(I_{i},f_{i},p_{i}\right)=I_{i}\cdot \left(E_{i}^{l},E_{i}^{r},E_{i}^{f}\right)=I_{i}^{l}E_{i}^{l}+I_{i}^{r}E_{i}^{r}+I_{i}^{f}E_{i}^{f}$$

where $E_{i}^{f}$ is the energy penalty when the computation task is failed. In this paper, the value of the energy penalty is set as the energy consumed by the maximum computation task. Here, the residual energy of $MD_{i}$ can be deduced by the equation above.

(10) $$E_{i}^{re}=B_{i}-E_{i}\left(I_{i},f_{i},p_{i}\right)$$

### 2.6. Reliability Model

In the MEC system described in this paper, the computation tasks can be executed locally or transmitted to the edge server for processing, while both of them will consume the energy stored in MDs, which is needed to ensure the reliability of MDs. In other words, MDs must support computation tasks executed locally or offloaded to the edge server successfully. The reliability model of MDs can be defined according to the description in [34].

**Definition** **1** (*Reliability of MDs*)**.** *Reliability of a mobile device refers to the probability of the MD working normally based on the energy consumption.*

With Definition 1, this paper assumes that the MD is reliable if the residual energy is greater than or equal to 0 after the computation task is accomplished successfully, and vice verse. In addition, the size of the computation task is subject to the uniform distribution of $0-S_{max}$. Therefore, joining Equations (9) and (10) and the distribution of the task size, the reliability of MDs (i.e., the probability of $MD_{i}$ working normally) can be obtained by substituting the offloading decision:

(11) $$\begin{array}{c} RP_{i}=Pr\left(E_{i}^{re}\geq 0\right)=Pr\left(B_{i}-E_{i}\left(I_{i},f_{i},p_{i}\right)\geq 0\right)\\ =\left\{\begin{array}{c} Pr\left(B_{i}\geq E_{i}^{l}\right)\;I_{i}^{l}=1\\ Pr\left(B_{i}\geq E_{i}^{r}\right)\;I_{i}^{r}=1 \end{array}\right.\\ =\left\{\begin{array}{c} Pr\left(S_{i}\leq \frac{B_{i}}{\kappa \;Qf_{i}^{2}}\right)\;I_{i}^{l}=1\\ Pr\left(S_{i}\geq \frac{B_{i}\omega \log_{2}\left(1+\frac{h_{i}p_{i}}{\sigma }\right)}{p_{i}}\right)\;I_{i}^{r}=1 \end{array}\right.\\ =\left\{\begin{array}{c} \frac{B_{i}}{\kappa \;S_{max}Qf_{i}^{2}}\;I_{i}^{l}=1\\ \frac{B_{i}\;\omega \;\log_{2}\left(1+\frac{h_{i}p_{i}}{\sigma }\right)}{p_{i}S_{max}})\;I_{i}^{r}=1 \end{array}\right. \end{array}$$

## 3. Problem Formulation

**Definition** **2** (*System Overhead*)**.** *System overhead refers to the weighted sum of the processing latency and energy consumption required to successfully execute a computation task.*

In this paper, the system overhead is used as a metric to evaluate the performance of offloading decisions for $MD_{i}$, i.e., how to process the computation task requested by $MD_{i}$. In the definition of the weighted sum, the weighted coefficient $\lambda^{t}$ is the preferred metric for process latency, and $\lambda^{e}$ is preferred for energy consumption. In addition, both of the coefficients should be satisfied by the equation $\lambda^{t}+\lambda^{e}=1$. Specifically, when the coefficient $\lambda^{e}$ of the system overhead is larger than $\lambda^{t}$, the energy consumption will be mainly considered. For this case, once the computation task is processed locally, a lower energy consumption means a longer working time of $MD_{i}$, which implies the battery life of $MD_{i}$ is prolonged. Conversely, for a delay-sensitive application, the processing latency coefficient $\lambda^{t}$ is larger to satisfy the requirement of the deadline. Therefore, the system overhead is used as a main metric for evaluating the performance of offloading decisions for the MEC system in this paper.

According to the definition above, combined with Equations (2) and (3), the system overhead of the computation task $T_{i}$ processed locally is

(12) $$ohd_{i}^{l}=\lambda^{t}L_{i}^{l}+\lambda^{e}E_{i}^{l}$$

Subsequently, joining Equations (6) and (7), the system overhead of the computation task $T_{i}$ transmitted to the edge server can be obtained by

(13) $$ohd_{i}^{r}=\lambda^{t}L_{i}^{r}+\lambda^{e}E_{i}^{r}$$

Additionally, the penalty for a failed computation task $T_{i}$ can be represented by

(14) $$ohd_{i}^{f}=\lambda^{t}L_{i}^{f}+\lambda^{e}E_{i}^{f}$$

In general, the system overhead of $MD_{i}$ in the MEC system to process the computation task can be expressed as

(15) $$\begin{array}{c} sys\_overhead_{i}=I_{i}\cdot \left(ohd_{i}^{l},ohd_{i}^{r},ohd_{i}^{f}\right)\\ =I_{i}^{l}ohd_{i}^{l}+I_{i}^{r}ohd_{i}^{r}+I_{i}^{f}ohd_{i}^{f} \end{array}$$

In summary, the computation task offloading in an MEC system of the Industrial Internet can be formulated as an MINLP, i.e., the cumulative sum of the system overhead of computation tasks requested by $MD_{i}$. The formulation of the problem is

(16) $$\begin{array}{c} \begin{array}{cc} \; & P1:\;\underset{\left(I_{i},f_{i},p_{i}\right)}{arg}min\sum_{i=1}^{N}sys\_overhead_{i}\\ \; & \begin{array}{cc} s.t. & C1:0<RP_{i}\leq 1\;i\in N\\ \; & C2:0\leq p_{i}\leq p_{i}^{max}\;i\in N\\ \; & C3:0\leq f_{i}\leq f_{i}^{max}\;i\in N\\ \; & C4:L_{i}\left(I_{i},f_{i},p_{i}\right)\leq D_{i}\;i\in N\\ \; & C5:|I_{i}|=1\;i\in N \end{array} \end{array} \end{array}$$

where *C*1 indicates that $MD_{i}$ should be reliable to support the execution of the computation task. *C*2 and *C*3 ensure that the transmission power and CPU frequency of $MD_{i}$ are within the specified range with the corresponding offloading decision, respectively. Besides these, the deadline of the computation task is also an important factor, and *C*4 gives the constraint of the deadline, i.e., the computation task required by $MD_{i}$ should be completed within the specified time, whether executed locally or offloaded to the edge server. Finally, *C*5 shows that the offloading decision is a 0–1 indicator.

## 4. Problem Solving and Algorithm Designing

### 4.1. Problem Solving

Clearly, the formulated problem P1 is an MINLP, which could be solved by the alternative optimization (AO) method, i.e., obtaining the optimal CPU cycle frequency $f_{i}^{*}$ for executing locally and transmitting the power $p_{i}^{*}$ for offloading to the edge server by setting the offloading decision while determining the final offloading decision according to the comparison results of the overhead consumed by different offloading decisions. Subsequently, we will obtain the optimal solution for the objective function. Since the computation task required by $MD_{i}$ can only be processed locally with the optimal CPU cycle frequencies or offloaded to the edge server with the optimal transmission power, different optimization variables, such as $f_{i}$ and $p_{i}$ in objective function, are independent from each other. Meanwhile, the offloading decision of each MD is constrained by the number of wireless channels existing in MDs and the edge server. Therefore, the problem P1 can be divided into two independent sub-problems to solve, i.e., the sub-problem related to the CPU cycle frequency for executing locally $P_{LO}$ and the sub-problem about the transmission power for offloading to the edge server $P_{CO}$.

#### 4.1.1. Optimal CPU Cycle Frequency

The sub-problem of the CPU cycle frequency for executing locally can be obtained by substituting $I_{i}^{l}=1$ and (2) and (3) into (16), i.e.,

(17) $$\begin{array}{c} \begin{array}{cc} \; & P_{\mathrm{LO}}:\;\underset{f_{i}}{arg}min\sum_{i=1}^{N}sys\_overhead_{i}\\ \; & \begin{array}{cc} s.t. & C1:0<RP_{i}=\frac{B_{i}}{\kappa \;S_{max}Qf_{i}^{2}}\leq 1\;i\in N\\ \; & C3:0<f_{i}\leq f_{i}^{max}\;i\in N\\ \; & C4:L_{i}\left(I_{i},f_{i},p_{i}\right)=\frac{S_{i}Q}{f_{i}}\leq D_{i}\;i\in N \end{array} \end{array} \end{array}$$

where

(18) $$\begin{array}{c} \begin{array}{cc} sys\_overhead_{i} & =ohd_{i}^{l}=\lambda^{t}L_{i}^{l}+\lambda^{e}E_{i}^{l}\\ & =\lambda^{t}\frac{S_{i}\;Q}{f_{i}}+\lambda^{e}\kappa \;S_{i}Qf_{i}^{2} \end{array} \end{array}$$

Since the local computing CPU cycle frequencies of each MD do not interfere with each other, the cumulative sum of this sub-problem can be decomposed into the sum of N minimums, that is, only the optimal $f_{i}$ of each MD needs to be calculated ($f_{i}$ is optimal when the execution overhead of the local process is the smallest). According to these, we express the objective function as $F\left(f_{i}\right)=sys\_overhead_{i}$, which is convex because both terms of $F(f_{i})$ are convex [35]. Meanwhile, by calculating the constraints *C*1, *C*3, and *C*4 in $P_{LO}$, the range of $f_{i}$ can be obtained. Specifically, the upper bound is $f_{i}^{max}$, while the lower bound is represented as follows:

(19) $$\begin{array}{c} \begin{array}{cc} \; & f_{i}^{min}=max\left\{\frac{S_{i}Q}{D_{i}},\sqrt{\frac{B_{i}}{\kappa \;S_{max}Q}}\right\} \end{array} \end{array}$$

Furthermore, a minimum exists when $F(f_{i})$ has a local minimum in the field of $f_{i}$ as it is a unimodal function. For the objective function $F(f_{i})$, $f_{i}^{0}={\left(\frac{\lambda^{t}}{2(1-\lambda^{t})\kappa }\right)}^{\frac{1}{3}}$ is the critical point, which can be obtained by solving the first derivative. Therefore, the monotonicity of $F(f_{i})$ can be analyzed according to the relationship between $f_{i}^{0}$ and the bounds of the domain. Firstly, the first derivative is always positive in [$f_{i}^{min},f_{i}^{max}$] when $f_{i}^{0}$ is smaller than $f_{i}^{min}$; therefore, $F(f_{i})$ is monotonically increasing in the domain of $f_{i}$. Then, in the same way, the objective function $F(f_{i})$ is monotonically decreasing when $f_{i}\in f_{i}^{min},f_{i}^{max}$ with $f_{i}^{0}$ is larger than $f_{i}^{max}$. Correspondingly, as the first derivative of $F(f_{i})$ is negative first and then positive when $f_{i}^{0}$ is between $f_{i}^{min}$ and $f_{i}^{max}$, the objective function first decreases and then increases.

Based on the monotonicity of the objective function $F(f_{i})$ above, the optimal CPU cycle frequency $f_{i}^{*}$ can be obtained by the closed form if and only if $f_{i}^{min}\leq f_{i}^{max}$:

(20) $$\begin{array}{c} f_{i}^{*}=\left\{\begin{array}{cc} f_{i}^{min}\; & f_{i}^{0}<f_{i}^{min}\\ f_{i}^{0}\; & f_{i}^{min}\leq f_{i}^{0}\leq f_{i}^{max}\\ f_{i}^{max}\; & f_{i}^{0}>f_{i}^{max} \end{array}\right. \end{array}$$

#### 4.1.2. Optimal Transmission Power

In the case of processing the computation task at the edge server, by substituting the variable of the offloading decision $I_{i}^{r}=1$ into the objective function of P1, we can obtain a new sub-problem about the optimal transmission power, i.e.,

(21) $$\begin{array}{c} \begin{array}{cc} \; & P_{\mathrm{CO}}:\;\underset{p_{i}}{arg}min\sum_{i=1}^{N}sys\_overhead_{i}\\ \; & \begin{array}{cc} s.t. & C1:0<RP_{i}=\frac{B_{i}v_{i}}{p_{i}S_{max}}\leq 1\;i\in N\\ \; & C2:0<p_{i}\leq p_{i}^{max}\;i\in N\\ \; & C4:L_{i}\left(I_{i},f_{i},p_{i}\right)=\frac{S_{i}}{v_{i}}\leq D_{i}\;i\in N \end{array} \end{array} \end{array}$$

in which, the objective function can be obtained by combing Equations (6), (7) and (13), that is

(22) $$\begin{array}{c} \begin{array}{cc} sys\_overhead_{i} & =ohd_{i}^{r}=\lambda^{t}L_{i}^{r}+\lambda^{e}E_{i}^{r}\\ & =\lambda^{t}\frac{S_{i}}{v_{i}}+\lambda^{e}\frac{p_{i}S_{i}}{v_{i}} \end{array} \end{array}$$

It can be found that the transmission powers of MDs are independent from each other, and there is no coupling. Thus, the minimum of the cumulative sum in sub-problem $P_{CO}$ can be decomposed into the sum of *N* minimums which will be the objective problem that needs to be solved. For convenience, the objective function can be denoted as $P(p_{i})$, which is convex, as discriminated by [36]. However, in Equation (21), both C1 and C4 are complex inequalities about $p_{i}$. Specifically, C1 is a fractional function, where the denominator is essentially a logarithmic function of $p_{i}$. Similarly, C4 comprises a logarithmic function. Therefore, the upper and lower bounds of $p_{i}$ in C1 and C4 are difficult to determine. To address this problem, we firstly obtain the bounds of the logarithmic function with $g(p_{i})$ as the following definition.

**Definition** **3.** *Combining (4), by denoting the function of $p_{i}$ as*

(23) $$g\left(p_{i}\right)=\frac{p_{i}}{v_{i}}=\frac{p_{i}}{\omega \log_{2}\left(1+\frac{h_{i}p_{i}}{\sigma }\right)}\;p_{i}>0$$

*the value range of*$g(p_{i})$*is* ($\sigma ln2{\left(\omega h_{i}\right)}^{-1},+\infty$).

**Proof.**  Since $g(p_{i})$ is monotonically increasing when $p_{i}>0$, its minimum value can be calculated by $\lim_{p_{i}\to 0}g\left(p_{i}\right)=\sigma ln2{\left(\omega h_{i}\right)}^{-1}$. The process of calculating, in detail, is relatively simple, and it is omitted here. □

According to the analysis above, the domain of $P(p_{i})$ can be determined, that is, the transmission power is not allowed beyond the maximum $p_{i}^{max}$, while the lower bound can be deduced by the initial battery capacity.

(24) $$\begin{array}{c} p_{i}^{min}=\left\{\begin{array}{cc} max\{p_{i,D_{i}},p_{i,B_{i}}\}\; & \frac{\sigma ln2\cdot \;S_{max}}{\omega h_{i}}\geq B_{i}\\ p_{i,D_{i}}\; & \frac{\sigma ln2\cdot \;S_{max}}{\omega h_{i}}<B_{i} \end{array}\right. \end{array}$$

where $p_{i,D_{i}}=\left(2^{\frac{S_{i}}{\omega D_{i}}}-1\right)\sigma /h_{i}$, and $p_{i,B_{i}}$ is the unique solution for $p_{i}S_{max}=B_{i}v_{i}$.

Similar to the analysis of the optimal CPU cycle frequency in the previous section, we can obtain the monotonicity of $P(p_{i})$, which is closely related to the critical point $p_{i}^{0}$. Therefore, as $P(p_{i})$ is a single variable function defined on [$p_{i}^{min},p_{i}^{max}$], the optimal solution of $p_{i}$ is given if and only if $p_{i}^{min}\leq p_{i}^{max}$.

(25) $$\begin{array}{c} p_{i}^{*}=\left\{\begin{array}{cc} p_{i}^{min}\; & p_{i}^{0}<p_{i}^{min}\\ p_{i}^{0}\; & p_{i}^{min}\leq p_{i}^{0}\leq p_{i}^{max}\\ p_{i}^{max}\; & p_{i}^{0}>p_{i}^{max} \end{array}\right. \end{array}$$

where $p_{i}^{0}$ is the unique solution for $\frac{dP(p_{i})}{dp_{i}}=0$. The specific expression of the equation is shown in (26), and it is proved to be a transcendental equation.

(26) $$\begin{array}{cc} \frac{dP(p_{i})}{dp_{i}}= & \frac{d(\lambda^{t}\frac{S_{i}}{v_{i}}+\lambda^{e}\frac{p_{i}S_{i}}{v_{i}})}{dp_{i}}\\ = & \frac{\left(1-\lambda^{t}\right)S_{i}\log_{2}\left(1+\frac{h_{i}p_{i}}{\sigma }\right)-\left[\lambda^{t}S_{i}+\left(1-\lambda^{t}\right)p_{i}S_{i}\right]\frac{h_{i}}{(\sigma +h_{i}p_{i})\ln2}}{\omega \log_{2}{\left(1+\frac{h_{i}p_{i}}{\sigma }\right)}^{2}} \end{array}$$

#### 4.1.3. Optimal Offloading Decision

Since the number of wireless channels is less than the number of MDs, the edge server does not provide a service for all computation tasks requested by MDs simultaneously. Thus, MDs should choose the offloading scheme for computation tasks based on the system overhead consumed by different execution modes under the reliability constraint. Meanwhile, the offloading scheme should satisfy the constraint of wireless channels, which would be implemented by the greedy policy. In more detail, if there exists an idle wireless channel, the greedy strategy is used to select the computation tasks with a lower system overhead to process at the edge server, i.e., $I_{i}^{l}=1$; otherwise, the computation tasks could only be executed locally, i.e., $I_{i}^{r}=1$. However, if the MD is not reliable, the computation task is viewed as a fail, namely, $I_{i}^{f}=1$, and its execution overhead is the penalty of latency and energy.

### 4.2. Algorithm Designing

The specific algorithm for solving the problem P1 is shown in Algorithm 1.

In this algorithm, the traversal of all MDs is executed firstly to determine the offloading scheme for MDs whose optimal CPU cycle frequency is 0. Then, computation overheads of all MDs executed by offloading computing are sorted in ascending order. When there are idle channels in the *M* wireless channels, MDs with the smallest system overhead in the ordered sequence and the offloading computing overhead, which is less than the local computing overhead, are selected for offloading computation, namely, the offloading scheme is $I_{i}^{m}=1$. However, when all the wireless channels are occupied, the offloading scheme is local computation. In summary, given that in the entire algorithm, all MDs are traversed twice, it can be gathered that the time complexity of Algorithm 1 is O(2N).
**Algorithm 1:** Heuristic Algorithm based on Greedy Policy for Task Offloading (HAGP)
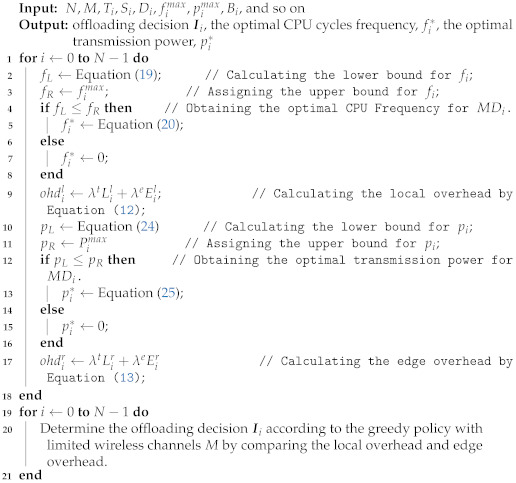


## 5. Simulation Results

### 5.1. Simulation Settings

Subsequently, we will verify the performance of HAGP with various simulation experiments. For convenience, some values of significant parameters are given in Table 2. As MDs are heterogeneous, the maximum of the CPU cycle frequency and the initial battery capacity are different and obey a uniform distribution in the value range. Furthermore, to illustrate the impact of different system parameters on the performance of the overall MEC system, we will show several simulation results by comparing with baseline offloading algorithms.

In addition, it can be found that the scenarios and objectives studied in this paper are different from the existing representative algorithms for computation task offloading with reliability, which are listed in Table 3. Thus, we compare HAGP with several baseline algorithms under the same conditions as follows:

1. **Local Computing All (LCA).** This means all the computation tasks generated by MDs are processed locally, which will not cause an overhead of the communication and computation on the edge server.
2. **Randomly Offloading Computing (ROC).** In this case, computation tasks requested by MDs are considered to be processed locally or offloaded to the edge server for completion. The offloading decision of each MD can be presented as a binary number, which is generated randomly.
3. **ALL Offloading Computing (AOC).** The algorithm requires all computation tasks on the MDs to be offloaded to the edge server for processing, which would consume the energy of MDs to transmit the data included in the computation tasks and the time delay during the computation tasks’ completion.

### 5.2. Analysis of Simulation Results

**Figure 2:**
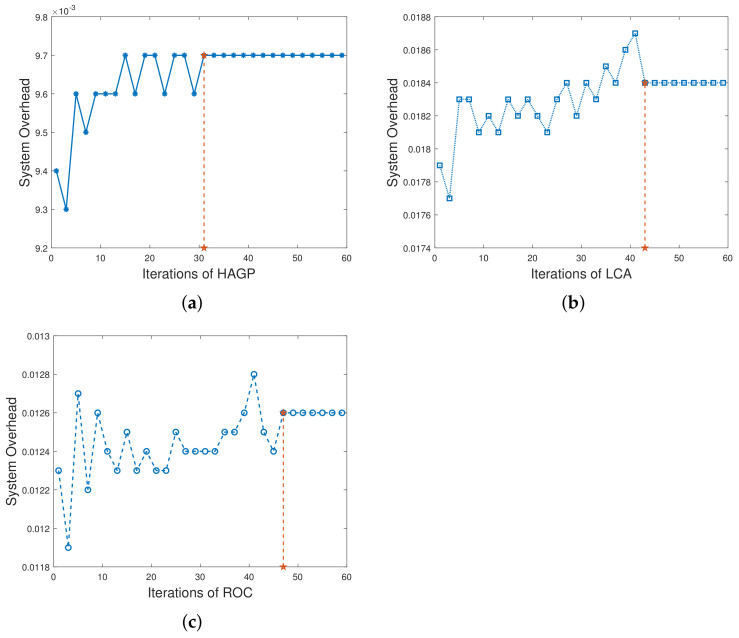
Overall system overhead vs. iterations of all three algorithms. (**a**) HAGP; (**b**) LCA; (**c**) ROC.

**Figure 3:**
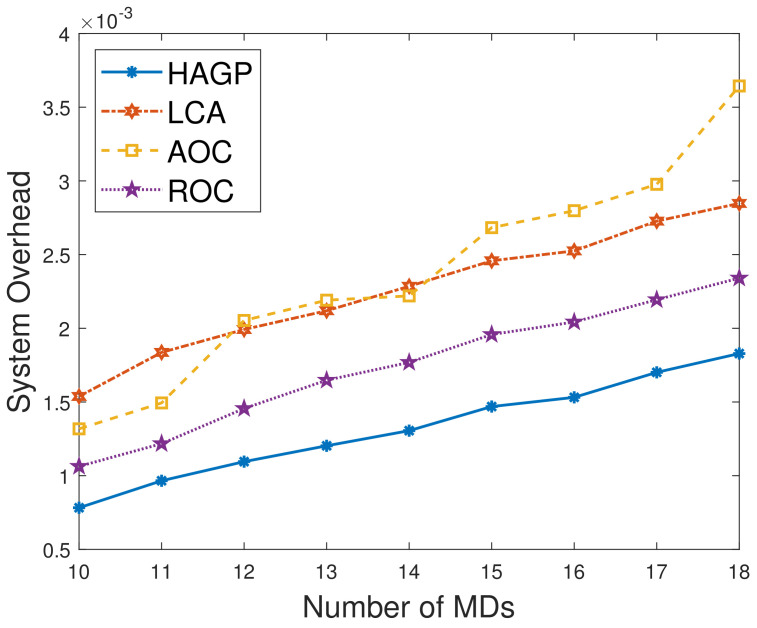
Overall system overhead vs. the number of MDs.

**Figure 4:**
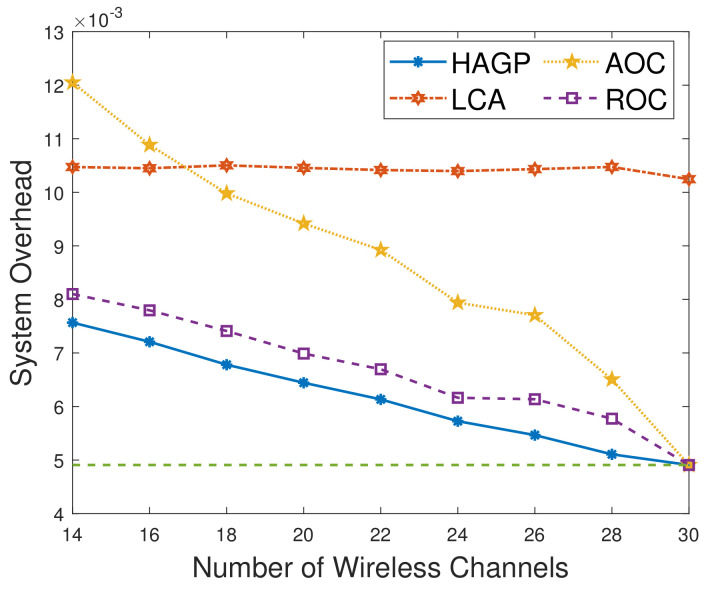
Overall system overhead vs. the number of wireless channels.

**Figure 5:**
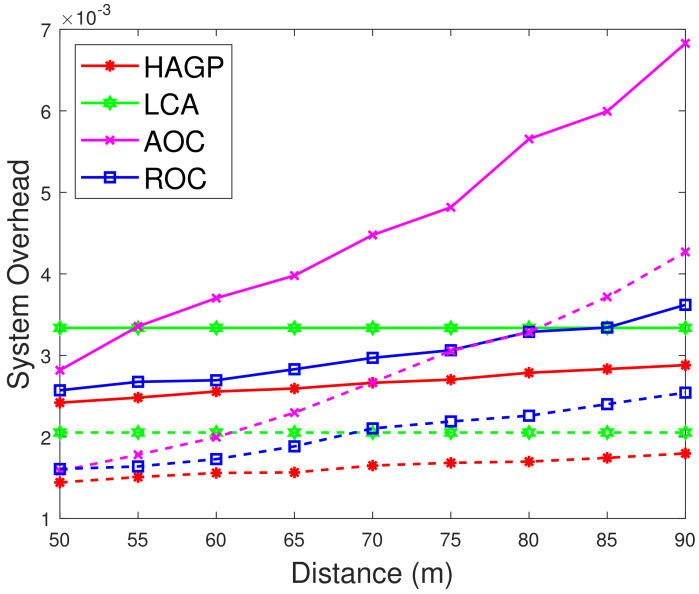
Overall system overhead vs. distance and $\lambda^{t}$. The solid curves represent $\lambda^{t}=0.8$, while the dash curves represent $\lambda^{t}=0.2$.

**Figure 6:**
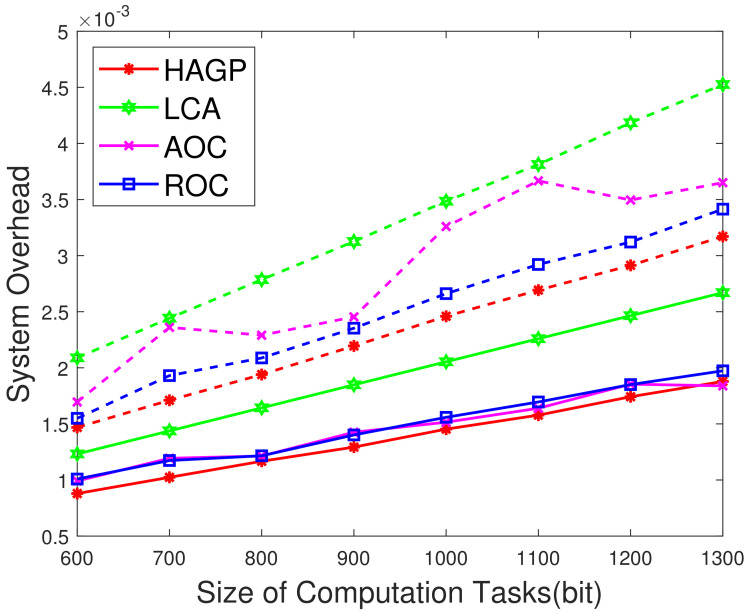
Overall system overhead vs. the size of computation tasks and $\lambda^{e}$. The solid curves represent $\lambda^{e}=0.8$, while the dash curves represent $\lambda^{e}=0.2$.

**Figure 7:**
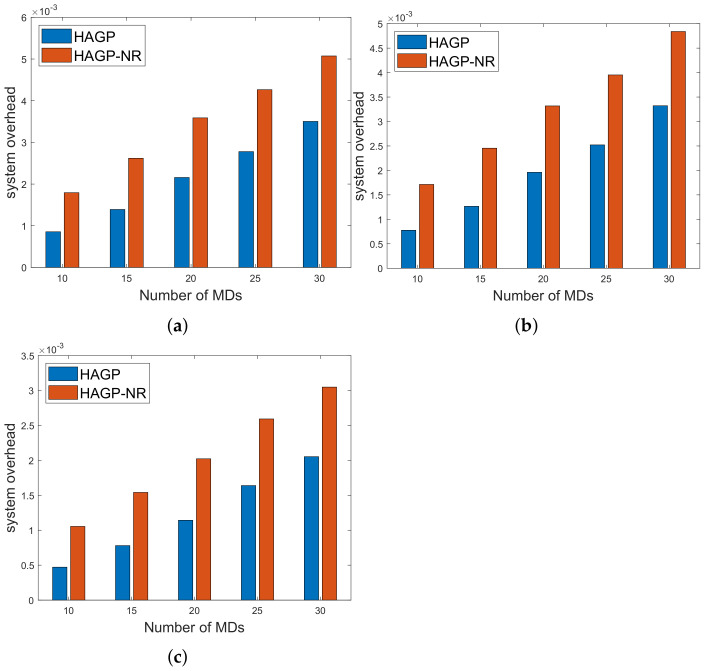
Comparison of HAGP and HAGP-NR. (**a**) $\lambda^{t}=0.8$; (**b**) $\lambda^{t}=0.5$; (**c**) $\lambda^{t}=0.2$.

(1) **The relationships of iterations and overall system overhead.** To ensure the simulation experiments are adaptable to different scenarios, some significant variables in this paper are given to obey a certain distribution, and MDs are heterogeneous. Therefore, to ensure the stability and accuracy, we define the overall system overhead as the average system overhead from multiple simulation results. As shown in Figure 2a, the overall system overhead of HAGP fluctuates with the number of iterations and converges from the 31st iteration. Similarly, it can be drawn from Figure 2b,c that the overall system overheads of LCA and ROC start to converge from the 43rd and 47th iterations, respectively. However, for AOC, the system overhead fluctuates within a very small range since the waiting time of computation tasks changes with the channel gain between MDs and the edge server. Therefore, for convenience, all the results of experiments in the paper adopt the average value of 50 iterations, which would satisfy the convergence of all algorithms.
(2) **Impact of the number of MDs on overall system overhead.** The relationship between the overall system overhead and the number of MDs is shown in Figure 3. It can be observed that, with the same simulation parameters given in Table 2, HAGP achieved the smallest overall system overhead compared with three baseline algorithms, including LCA, AOC, and ROC. This is because the computation task with the largest local execution overhead is chosen to be offloaded in HAGP, while the overhead consumed by offloading to the edge server is much smaller than that generated locally. Furthermore, when the number of wireless channels in the system remains unchanged, with the number of MDs increasing from 10 to 18, the overall system overhead becomes larger and larger in all algorithms. This is because the overall overhead of the system is closely related to the number of MDs in the system, that is, the more MDs, the more computation tasks it handles, and accordingly, the greater the overall system overhead.
(3) **Impact of the number of wireless channels on overall system overhead.** To illustrate the impact of the number of wireless channels on the overall system overhead, we set some system parameters included in the MEC system as follows: the number of MDs is 30, the size of the computation task is 1000 bit, the distance between MDs and the edge server is 50 m, the weighted coefficient of the time delay is 0.8, and the number of wireless channels ranges from 14 to 30. As presented in Figure 4, the overall system overhead in MEC decreases with the increasing number of wireless channels in several offloading algorithms, such as HAGP, AOC, and ROC, while it does not fluctuate too much in LCA. This is because the overall system overhead of LCA is irrelevant as the wireless channels for the computation tasks are all processed locally without transmitting data to the edge server. Thus, the overall system overhead is only decided by the heterogeneous computing capacity of MDs, which has a small value range listed in Table 2. However, the computing overhead consumed by the offloading computing model is much smaller than local processing; therefore, the greater the number of wireless channels, the more computation tasks will be offloaded, and the less the overall system overhead will be. Meanwhile, it can be found that when the number of wireless channels infinitely approaches the number of MDs, the overall system overhead converges to a fixed value.
(4) **Impact of distances and weighted coefficients on overall system overhead.**Figure 5 shows the effects of two different factors of the MEC system in this paper, including distances between MDs and the edge server and the weighted coefficient of the processing latency. To obtain the relationship between these two different factors and the system overhead accurately, we set other parameters to be fixed with 50 iterations. Firstly, we can see that when the weighted coefficients remain unchanged, the overall system overhead increases with the increasing distances for several offloading algorithms, including HAGP, RCA, and ROC, while it stays the same for LCA. This is because computation tasks are all processed locally, which is irrelevant to the location of MDs from the edge server, while the distances affect the channel gain between MDs and the edge server according to Equation (4), which determines the transmission rate of offloading tasks as an important component. Secondly, for three offloading algorithms with the same weighted coefficient, the overall system overhead of HAGP is always lower than the other two. At the same time, as the distances increase, the overall system overhead of AOC increases the most. By analyzing, it can be observed that AOC is mainly affected by the waiting latency of computation tasks for limited wireless channels, while HAGP and ROC can be chosen to execute locally. Finally, for all algorithms, the overall system overhead with a coefficient equal to 0.8 is higher than that with 0.2. The reason is that the weighted coefficient represents the proportion of time latency in the overall system overhead, while the distances are closely related to the time latency. Therefore, the weighted coefficient is larger, and the overall system overhead is higher.
(5) **Impact of computation task size and weighted coefficients on overall system overhead.** According to (11), it can be found that the reliability of MDs is inversely proportional to the maximum size of the computation tasks. Therefore, we conducted many simulation experiments with different maximum sizes of the computation task, ranging from 600 to 1300 (bits). As described in Figure 6, the overall system overhead increases with the increasing maximum size of the computation tasks. This is because, as the maximum size of the computation tasks increases, the reliability of the MDs will decrease. At this time, the probability of the task being re-requested or discarded will increase, and accordingly, the overall system overhead will increase. In addition, when $\lambda^{e}=0.8$, the energy consumption is a metric paid more attention in the system overhead. Therefore, the size of computation tasks is considered to show a decreasing relationship between the system overhead and the weighted coefficient of energy consumption. In other words, when $\lambda^{e}$ decreases, the system overhead increases, which is consistent with Figure 5. In addition, it is observed that HAGP will obtain the minimal overall system overhead compared with the other classical algorithms under the same maximum size of computation tasks.
(6) **Comparison of HAGP and HAGP without considering the reliability of MDs.** In this paper, the authors studied task offloading with the reliability of MDs for MEC in the Industrial Internet. Therefore, the impact of the reliability of MDs on the system overhead is an important metric to certify the performance of HAGP. As shown in Figure 7, the comparisons of HAGP and HAGP without considering the reliability of MDs (termed as HAGP-NR) with different weighted coefficients are listed. Obviously, the overall system overhead of HAGP is lower than HAGP-NR in all figures, including Figure 7a–c, where the weighted coefficient is 0.8, 0.5, and 0.2, respectively. This is because for HAGP, it can determine whether the MD is reliable before the task is executed, i.e., when the MD is reliable, it is performed and causes the system overhead; otherwise, it is not performed. However, for HAGP-NR, the computation tasks are processed regardless of whether the MD is reliable. At this time, once the MD is unreliable, the task being executed will not only be disrupted and discarded but will also consume a little more system overhead than HAGP, that is, no matter whether the MD is reliable to process the computation task, the system overhead will be incurred. In a nutshell, compared with HAGP-NR, HAGP can save the corresponding system overhead by judging the reliability of the MD. In addition, since $\lambda^{e}$ is the weighted coefficient of energy consumption in the system overhead, only the total value of the system overhead in all three figures changes, and the comparison trend of HAGP and HAGP-NR does not change.

## 6. Conclusions

To make the offloading scheme adaptive to an uncertain mobile environment, and to minimize the system overhead of MEC, this paper considered the reliability of MDs and proposed a heuristic algorithm based on greedy policy for task offloading in an MEC system of the Industrial Internet, namely, HAGP. By constructing different computing models and formulating the objective function, we obtained a mixed integer non-linear programming problem and achieved the optimal solution by elementary mathematics methods. Meanwhile, we determined the optimal offloading decision for each MD which can be verified by comparing several baseline algorithms with extended simulations. In addition, the paper explains the effect of several key factors in the MEC system on the system overhead, such as the distance between MDs and the edge server, the weighted coefficient of time latency and energy consumption, and the computation task size. Finally, by comparing with HAGP-NR, it can be found that HAGP can effectively save the system overhead by judging the reliability of MDs, which will further prolong the battery life of MDs and support more computation tasks.

Based on the ideas in this paper, there are some limitations that need to be studied in future works. Specifically, (1) to handle the interdependent computation tasks within the deadline, the buffer will be considered in the model; (2) to explore the reliability of communication, the re-transmission and cooperation will be focused on; (3) to minimize the cost of the offloading scheme, the energy consumption of processing tasks at the edge side should be considered.

## Figures and Tables

**Table 1:** Important symbols used in the paper and their description.

Symbols	Description
N(N)	The set of MDs (the number of elements in set)
M(M)	The set of wireless communication channels (the number of elements in set)
*Q*	The number of CPU cycle frequency for processing one bit data
$T_{i}$	The computation task requested by $MD_{i}$
$S_{i}$($S_{max}$)	The (maximum) size of the computation task requested by $MD_{i}$ (in bit)
$D_{i}$	The deadline of the computation task $T_{i}$ (in ms)
$I_{i}^{m}$	The indicator of whether the computation task on $MD_{i}$ is offloaded, where m∈{l,r,f}
$d_{i}$	The distance between $MD_{i}$ and the edge server (in m)
$h_{i}$	The channel gain between $MD_{i}$ and the edge server during the transmission of the computation task
$f_{i}$($f_{i}^{max}$)	The (maximum) frequency of $MD_{i}$ to process the computation task locally (in Hz)
$p_{i}$($p_{i}^{max}$)	The (maximum) transmission power of $MD_{i}$ to transmit the computation task (in w)
$L_{i}^{m}$	The execution latency of the computation task $T_{i}$, where m∈{l,r,f} (in ms)
$B_{i}$	The battery capacity of $MD_{i}$ (in J)
$E_{i}^{m}$	The energy consumption of the computation task $T_{i}$, where m∈{l,r,f} (in J)

**Table 2:** Parameters and values.

Parameter	Value	Parameter	Value
$f_{i}^{max}$	[0.8,1.9] (GHz)	ω	1 (MHz)
$S_{i}^{max}$	1000 (bit)	Q	737.5 (CPB)
$p_{i}^{max}$	1 (W)	σ	$10^{-13}$ (W)
$B_{i}$	[$5.5\times \;10^{-6},10^{-5}$] (J)	κ	$10^{-28}$
$D_{i}$	0.002 (ms)	$g_{0}$	−40 (dB)
$d_{i}$	(0,50] (m)	$\lambda^{t}\left(\lambda^{e}\right)$	{0.2,0.5,0.8}
$L_{i}^{f}$	0.002 (ms)	$E_{i}^{f}$	0.001 (mJ)

**Table 3:** Differences between several algorithms.

Algorithms	Number of MDs	Number of Edge Servers	Reliability	Objective Function
RLT-based [19]	1	*N*	transmission reliability	product of total latency and the transmission reliability
DLRAP [20]	*N*	*M*	reliability of tasks	the energy consumption of computing and transmission
EASE [21]	*N*	*M*	reliable computing mode	the energy consumption of the system
**HAGP**	*N*	1	reliability of MDs	the weighted sum of time delay and energy consumption

## Data Availability

Data sharing is not applicable to this article.
